# Prevalence of and Short-term Changes in Conjunctival Manifestations Among Patients With SARS-CoV-2 Infection

**DOI:** 10.1001/jamanetworkopen.2022.7734

**Published:** 2022-04-19

**Authors:** Emilio Pedrotti, Erika Bonacci, Marco Ligozzi, Jacopo Bonetto, Raphael Kilian, Davide Gibellini, Giorgio Marchini

**Affiliations:** 1 Ophthalmic Unit, Department of Neurosciences, Biomedicine, and Movement Sciences, University of Verona, Verona, Italy; 2 Microbiology and Virology Section, Department of Diagnostic and Public Health, University of Verona, Verona, Italy; 3 Ophthalmic Unit, Hospital of Meran, Merano, Italy

## Abstract

This cohort study assesses the prevalence of SARS-CoV-2–related conjunctival manifestations and conjunctival swab positivity on hospital admission and 3 days thereafter for patients not receiving anti-inflammatory treatment.

## Introduction

Studies suggest that SARS-CoV-2 can infect the conjunctival mucosa.^1,2,3,4^ Signs of ocular involvement have been reported in 11% to 32% of SARS-CoV-2 cases, with a rate of conjunctival swab (CS) positivity from 0% to 57%. Previous studies lacked a predetermined sample size, relied on questionnaires and/or interviews, or analyzed nonhospitalized and hospitalized patients receiving anti-inflammatory agents. In this study, we assessed the prevalence of SARS-CoV-2–related conjunctival manifestations and CS positivity on hospital admission (T1) and 3 days thereafter (T2) for a predetermined sample of patients not receiving anti-inflammatory treatment.

## Methods

This cohort study was approved by the ethics committees of 3 tertiary hospitals (Hospital of Verona, Hospital of Meran, and Hospital of Bozen). Participants provided written informed consent. The study followed the STROBE reporting guideline.

We enrolled hospitalized patients with SARS-CoV-2 infection who (1) had not received previous or concomitant antiviral or corticosteroid treatment or any topical ocular treatment, (2) had no eyelid pathologies, and (3) were not receiving continuous positive-airway pressure treatment. At T1 and T2, 2 ophthalmologists performed an ocular surface examination (OSE) and CS sampling of both eyes separately. Conjunctival redness (CR) was graded with the Efron scale; other conjunctival signs and symptoms (OCSSs) were considered together. Real-time polymerase chain reaction was used to confirm SARS-CoV-2 infection and exclude other conjunctival pathogens.

After T1, all patients received hyaluronic acid artificial tears (ATs) twice daily in the clinically worse eye. Nonpreservative-free (NPF) ATs with povidone iodine (Medivis) were administered at 1 hospital and preservative-free (PF) ATs (SIFI) were administered at 2 hospitals. We used the McNemar-Bowker test to compare CR and OCSSs between T1 and T2 and between AT-treated and nontreated eyes. We used a Fisher exact test to compare CS positivity and OSE for the NPF- and PF-AT formulations. Multivariate analysis was performed using ordinal logistic regression. *P* < .05 was considered significant.

## Results

Of the 144 patients enrolled, 2 had a positive CS result only for bocavirus and parainfluenza at T1 and were excluded. Of the 142 patients evaluated, 92 (64.8%) were men and 50 (35.2%) were women (mean [SD] age, 71 [18.2] years).

Eighteen patients (12.7%) had a positive CS result; 64 (45.1%) had at least 1 clinical finding (eg, CR, most commonly grade 2) (Table 1). The CR grade was significantly higher in eyes with CS positivity (*P* < .001).

**Table 1.** Clinical Findings and SARS-CoV-2 Conjunctival Swab Positivity at Baseline and After 3 Days for Eyes Treated With Artificial Tears and Untreated Eyes

Characteristic	No. of eyes (%)		*P* value
	T1	T2	
AT treated			
Conjunctival redness score^a^			
0	78 (54.9)	104 (73.2)	<.001
1	18 (12.7)	31 (21.8)	
2	36 (25.4)	7 (5.0)	
3	7 (4.9)	0	
4	3 (2.1)	0	
No. of OCSSs^b^			
0	79 (55.6)	124 (87.3)	<.001
1	50 (35.2)	17 (12.0)	
2	13 (9.1)	1 (0.7)	
Conjunctival swab result			
Positive	16 (11.3)	2 (1.4)	<.001
Negative	126 (88.7)	140 (98.6)	
Nontreated			
Conjunctival redness score			
0	85 (59.9)	76 (53.5)	.002
1	27 (19.0)	50 (35.2)	
2	27 (19.0)	12 (8.5)	
3	3 (2.1)	4 (2.8)	
4	0	0	
No. of OCSSs			
0	97 (68.3)	99 (69.7)	.17
1	37 (26.0)	38 (26.8)	
2	8 (5.7)	5 (3.5)	
Conjunctival swab result			
Positive	5 (3.5)	10 (7.0)	.09
Negative	137 (96.5)	132 (93.0)	

Abbreviations: AT, artificial tear; OCSS, other conjunctival sign and symptom; T1, baseline; T2, after 3 days.

^a^ Conjunctival redness was measured with the Efron scale.

^b^ Ocular sign and symptoms included discharge, itching, chemosis, photophobia, tearing, or visual impairment.

At T2, 12 patients (8.5%) had a positive CS result (Table 2). There were 5 additional patients in the non-AT group (*P* = .10) and 14 fewer patients in the AT group (*P* = .001). Redness worsened among nontreated eyes (*P* = .001), whereas both CR and OCSSs improved among AT-treated eyes (both *P* < .001). Compared with PF-ATs, these results suggest that NPF-ATs were 1.13 times more effective at improving CR and were the only formulation in this study able to reduce OCSS and CS positivity.

**Table 2.** Clinical Findings and SARS-CoV-2 Conjunctival Swab Positivity at Baseline and After 3 Days in Eyes Treated With Artificial Tears and Untreated Eyes, Considering Preservative-Free and Nonpreservative-Free Formulations Separately

Characteristic	Eyes treated with PF-ATs (n = 39)			Eyes treated with NPF-ATs (n = 103)		
	T1, No. (%)	T2, No. (%)	*P* value	T1, No. (%)	T2, No. (%)	*P* value
AT treated						
Conjunctival redness score^a^						
0	19 (48.8)	21 (53.9)	.06	59 (57.3)	83 (80.6)	<.001
1	9 (23.0)	14 (35.9)		9 (8.7)	17 (16.5)	
2	11 (28.2)	4 (10.2)		25 (24.3)	3 (2.9)	
3	0	0		7 (6.8)	0	
4	0	0		3 (2.9)	0	
No. of OCSSs^b^						
0	26 (66.7)	29 (74.4)	.37	53 (51.5)	95 (92.2)	<.001
1	12 (30.8)	9 (23.1)		38 (36.9)	8 (7.8)	
2	1 (2.5)	1 (2.2)		12 (11.6)	0	
Conjunctival swab result						
Positive	4 (10.3)	2 (5.1)	.06	12 (11.7)	0	<.001
Negative	35 (89.7)	37 (94.9)		91 (88.3)	103 (100)	
Nontreated						
Conjunctival redness score						
0	19 (48.8)	21 (53.9)	.03	66 (64.1)	55 (53.4)	.002
1	12 (30.8)	15 (38.5)		15 (14.6)	35 (34.0)	
2	8 (20.4)	3 (7.6)		19 (18.4)	9 (8.7)	
3	0	0		3 (2.9)	4 (3.9)	
4	0	0		0	0	
No. of OCSSs						
0	28 (71.9)	29 (74.4)	>.99	69 (67.0)	70 (68.0)	>.99
1	10 (25.6)	9 (23.1)		27 (26.2)	29 (28.1)	
2	1 (2.5)	1 (2.2)		7 (6.8)	4 (3.9)	
Conjunctival swab result						
Positive	3 (7.7)	3 (7.7)	>.99	2 (1.9)	7 (6.8)	.63
Negative	36 (92.3)	36 (92.3)		101 (98.1)	96 (93.2)	

Abbreviations: AT, artificial tear; OCSS, other conjunctival sign and symptom; NPF, nonpreservative free; PF, preservative free; T1, baseline; T2, after 3 days.

^a^ Conjunctival redness was measured with the Efron scale.

^b^ Ocular signs and symptoms included discharge, itching, chemosis, photophobia, tearing, or visual impairment.

## Discussion

The variability of prevalence data in the literature might be due to a lack of predetermined samples and wide inclusion criteria. We applied strict inclusion criteria and assessed the sample size and found that the prevalence of conjunctival manifestations and CS positivity among patients with SARS-CoV-2 was 45.1% and 12.7%, respectively. This discrepancy could be associated with the inflammatory response related to systemic viral infections. The most frequent conjunctival manifestation was CR, a key sign of viral conjunctivitis. However, because OSE was performed at the patient bedside and other typical findings (eg, conjunctival membranes or pseudomembranes and follicles) were not assessed, a definitive diagnosis of viral conjunctivitis could not be made. Study limitations include restricted follow-up and a lack of definitive diagnoses.

These results suggest a concordance between CR grade and CS positivity. Although the difference in prevalence between the AT-treated groups could have affected the statistical analysis, our findings suggest that the virucidal activity of povidone iodine within NPF-ATs may be associated with conjunctival manifestation improvement and CS negativity among patients with SARS-CoV-2 infection.^5,6^
